# Vulvar lipoma: a case report

**DOI:** 10.1186/1752-1947-8-203

**Published:** 2014-06-18

**Authors:** Sofia Jayi, Meriem Laadioui, Hind El Fatemi, Fatima Zohra Fdili, Hakima Bouguern, Hikmat Chaara, Afaf Laamarti, My Abdelilah Melhouf

**Affiliations:** 1 Department of Gynecology and Obstetrics, University Hospital of Fez, Sidi Mohammed Ben Abdellah University, 37-39, Lotissement Asmae, Route Ain Chqef, Fez, Morocco; 2 Department of Anatomopathology, University Hospital of Fez, Sidi Mohammed Ben Abdellah University, 37-39, Lotissement Asmae, Route Ain Chqef, Fez, Morocco

**Keywords:** Diagnosis, Lipoma, Surgical excision, Vulva

## Abstract

**Introduction:**

Vulvar lipoma is a rare tumor localization and only a few cases have been reported. The clinical characteristics of vulvar lipoma are well known. However, it is important to distinguish lipomas from liposarcomas. We report a case of vulvar lipoma and discuss its clinical features, including diagnostic aspects, with emphasis on histopathological evaluation of all excised lesions. We also report and discuss patient management and treatment outcomes.

**Case presentation:**

We report the case of a 27-year-old Moroccan woman. Our patient presented with a painless and slow-growing right vulvar mass that had evolved over one year, which had suddenly become uncomfortable when walking. A physical examination revealed a single soft and pasty mass in her left labium majus, which could be mobilized under her skin towards her mons pubis. The largest dimension of the mass measured 6cm. Magnetic resonance imaging showed a homogenous hyperintense mass with a well-defined contour in her left labium majus; a fat-suppressed magnetic resonance image demonstrated a marked signal intensity decrease. The mass was completely removed surgically. A histological examination revealed a circumscribed benign tumor composed of mature adipocytes, confirming the diagnosis of vulvar lipoma.

**Conclusion:**

Vulvar lipomas must be differentiated from liposarcomas, which demonstrate very similar clinical and imaging profiles. The final diagnosis should be based on histopathological evaluation. A precise diagnosis should allow for appropriate surgical treatment.

## Introduction

Lipomas are widely disseminated benign mesenchymal neoplasms commonly found over the neck and upper back, shoulders, abdomen, buttocks, and proximal portions of the extremities [1]. Vulva localizations are rare, and very few cases have been reported [2,3]. In this paper, we report the case of a vulvar lipoma that was diagnosed in a 27-year-old woman. We discuss the clinical features and management options of this pathology with emphasis on the histopathological evaluation of all excised lesions. A review of the literature is also presented.

## Case presentation

**Figure 1:**
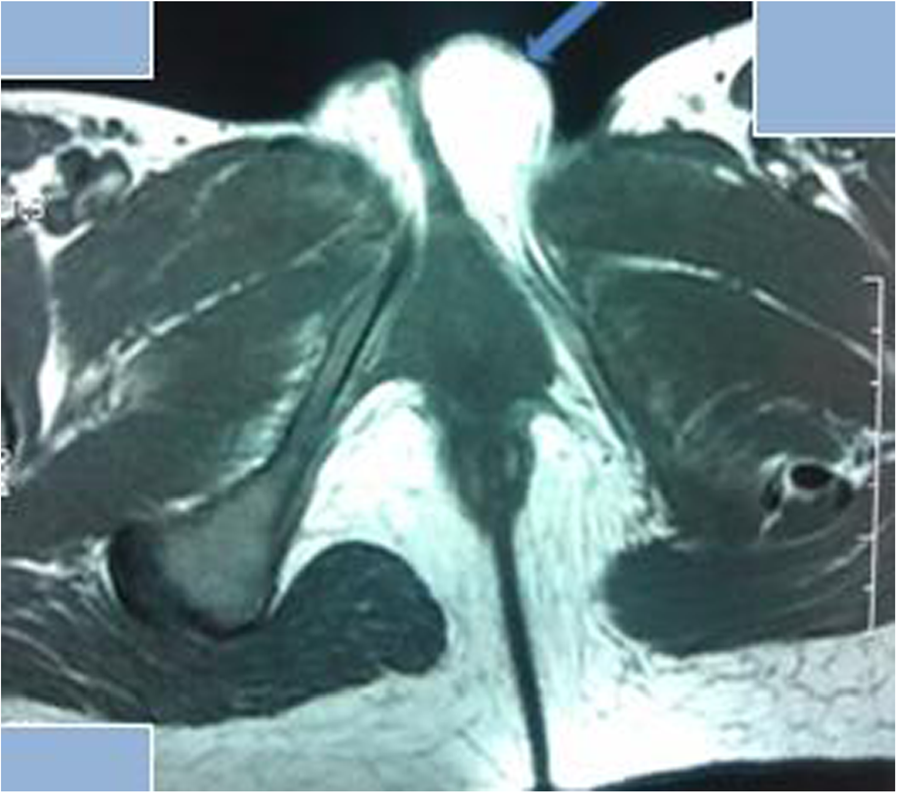
Axial T1-weighted magnetic resonance image shows a homogenous, hyperintense mass with a well-defined margin in the left labium majus (arrow).

**Figure 2:**
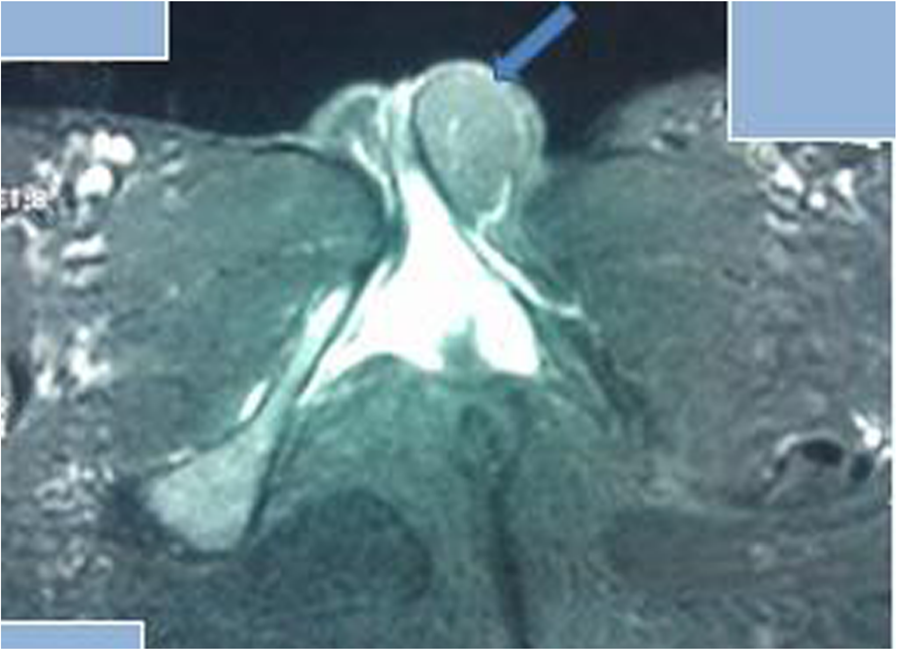
**Axial T2-weighted, fat-suppressed magnetic resonance image.** Markedly decreased signal intensity of the mass is found (arrow).

**Figure 3:**
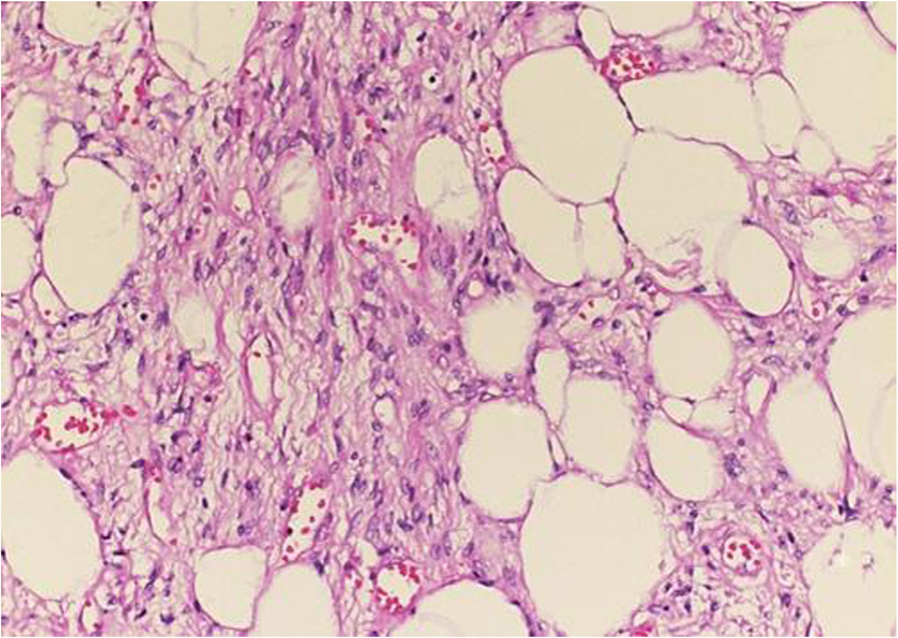
**Sections of tumor showing mature adipocytes organized into lobules separated by fibrous septa.** Hematoxylin and eosin stain ×10.

We report the case of a 27-year-old Moroccan woman, who presented with a painless and slow-growing right vulvar mass that evolved over one year. Our patient reported a sudden, uncomfortable feeling during walking. A physical examination revealed a single soft and pasty mass in her left labium majus. The mass could be mobilized under her skin towards her mons pubis. The largest dimension of the mass measured 6cm. However, there was no visible palpable cough impulse or inguinal lymphadenopathy, while a physical pelvic examination was normal.Magnetic resonance imaging (MRI) was performed (Figures 1 and 2), which demonstrated a homogenous and hyperintense mass with a well-defined contour in our patient’s left labium majus. Fat-suppressed MRI demonstrated a marked decrease in the mass signal intensity.Surgery allowed complete mass removal. A histologic study of the tumor slices showed lobulated yellow tissue without hemorrhage or necrosis. A microscopic examination revealed a circumscribed benign tumor composed of mature adipocytes, confirming the diagnosis of vulvar lipoma (Figure 3).

## Discussion

Lipomas are very common benign tumors in soft tissues derived from mesenchymal cells [2]. They have been identified in various age groups ranging from infancy to late-age decades [1]. They usually appear between 40 and 60 years of age [2]. Nevertheless, their precise etiology and pathogenesis not completely elucidated. Trauma seems to be implicated in some cases [1,4]. Our patient was in her third decade of life without any history of trauma.

Lipomas usually present as single or multiple painless slowly growing and mobile swelling soft tissue with a pasty characteristic. The tumor shows ill-defined, well-demarcated, or pedunculated aspects that are not adherent to the overlying skin. These characteristics allow correct diagnosis in most cases by clinical examination [1-6]. However, vulvar lipomas have to be differentiated from liposarcomas, which are rare but have a very similar clinical profile to lipoma, including cystic swellings of Bartholin’s gland and Nuck’s canal, and inguinal hernia, especially in children [1-3].

In developing countries, ultrasound is recommended instead of expensive imaging investigation because of its availability and cost-effectiveness [1]. Vulvar lipoma ultrasound demonstrates a non-specific homogenous echogenic mass with lobular structures consistent with fat deposition [1,6,7]. Computed tomography and MRI are useful for evaluating tumor extensions and anatomical connections with surrounding structures [2,6,7]. MRI is a supportive tool to differentiate vulvar lipomas from liposarcomas [1,3,8]. However, Ohguri *et al*. showed that septal enhancement in contrast-enhanced MRI allows the differentiation of liposarcoma [3]; they found moderate or marked septal enhancement in 25% and 75%, respectively, of well-differentiated liposarcomas [8]. Therefore, soft lipomatous lesions with thin septa that are not enhanced on MRI could be diagnosed as lipoma [3,9].

Treatment for a lipoma without excision includes steroid injection and liposuction and is common. However, complete surgical excision is the treatment of choice for vulvar lipoma [1]. Surgery also allows for excluding any malignant tumoral evolvement via a histological study [1,2]. Typically, a histological study shows a thin peripheral capsule surrounding a lobular proliferation of adipocytes [3]. Recurrence is possible; short-term recurrence should draw the attention of clinicians to possible malignant tumor evolvement. Indeed, authors have reported histological diagnosis difficulties that suggested a borderline adipose tumor [10].

## Conclusion

Vulvar lipomas need to be differentiated from liposarcomas, which demonstrate very similar clinical and imaging profiles. The final diagnosis should be based on histopathological evaluation. A precise diagnosis should allow for appropriate surgical treatment.

## Consent

Written informed consent was obtained from the patient for publication of this case report and any accompanying images. A copy of the written consent is available for review by the Editor-in-Chief of this journal.

## Competing interests

The authors declare that they have no competing interests.

## Authors’ contributions

SJ was the principal author and major contributor in writing the manuscript. ML reviewed the literature; HEF performed the histological study; AA participated in the histological study; FZF, HB and HC analyzed and interpreted the data from our patient; MAM corrected the manuscript. All authors read and approved the final manuscript.
